# Frailty and disability among older adults residing in Rohingya refugee camp in Bangladesh

**DOI:** 10.1371/journal.pone.0341499

**Published:** 2026-01-23

**Authors:** Afsana Anwar, Mahmood Parvez, Farhan Azim, Uday Narayan Yadav, Saruna Ghimire, Ateeb Ahmad Parray, Shovon Bhattacharjee, ARM Mehrab Ali, Rashidul Alam Mahumud, Md Irteja Islam, Md Nazmul Huda, Mohammad Enamul Hoque, Probal Kumar Mondal, Abu Ansar Md Rizwan, Suvasish Das Shuvo, Sabuj Kanti Mistry

**Affiliations:** 1 Discipline of Psychiatry and Mental Health, School of Clinical Medicine, University of New South Wales, Sydney, Australia; 2 Ministry of Health and Family Welfare, Government of Bangladesh, Dhaka, Bangladesh; 3 Melbourne Graduate School of Education, University of Melbourne, Melbourne, Australia; 4 National Centre for Epidemiology and Population Health, The Australian National University, Canberra, Australia; 5 International Centre for Future Health Systems, University of New South Wales, Sydney, Australia; 6 Department of Sociology and Gerontology and Scripps Gerontology Center, Miami University, Oxford, Ohio, United States of America; 7 Department of International Health, Johns Hopkins Bloomberg School of Public Health, Johns Hopkins University, Baltimore, United States of America; 8 ARCED Foundation, Dhaka, Bangladesh; 9 NHMRC Clinical Trials Centre, Faculty of Medicine and Health, The University of Sydney, Sydney, Australia; 10 School of Public Health, Faculty of Medicine and Health, The University of Sydney, Sydney, Australia; 11 Research, Innovation and Grants, Spreeha Bangladesh, Dhaka, Bangladesh; 12 Chapainawabganj Polytechnic Institute, Rajshahi, Bangladesh; 13 Department of Nutritional Sciences, Texas Tech University, Lubbock, Texas, United States of America; 14 Department of Nutrition and Food Technology, Jashore University of Science and Technology, Jashore, Bangladesh; 15 School of Medical, Indigenous, and Health Sciences, University of Wollongong, Woolongong, Australia; 16 School of Population Health, University of New South Wales, Sydney, Australia; 17 Department of Public Health, Daffodil International University, Dhaka, Bangladesh; Universidad Nacional Autonoma de Mexico, MEXICO

## Abstract

**Background:**

Frailty and disability often emerge with ageing and affect quality of life. Older adults residing in Rohingya refugee camp in Bangladesh are particularly susceptible to frailty and disability due to adverse physical and social environment along with limited health and social care services available in the camp. This study aimed to investigate the prevalence and factors associated with frailty and disability among Rohingya older adults living in Bangladesh.

**Methods:**

This cross-sectional study was conducted among older adults aged ≥60 years residing in the Rohingya refugee settlement in Bangladesh. The primary outcomes were frailty and disability, explored using the ‘Frail Non-Disabled (FiND) questionnaire. Data were collected face-to-face during November-December 2021, using a semi-structured questionnaire. A multinomial logistic regression model was used to identify the factors associated with frailty and disability.

**Results:**

The majority of participants (n = 864) were aged 60–69 years (72.34%), male (56.25%), married (79.05%), and without formal education (89.0%). The study revealed a high prevalence of frailty (36.92%) and disability (55.21%) among the participants. The multinomial regression analysis showed that the likelihood of experiencing disability was significantly higher among participants who were aged 70–79 years (RRR = 2.65, 95% CI: 1.25, 5.66) and ≥80 years (RRR = 8.06, 95% CI: 1.05, 61.80), were female (RRR = 3.93, 95% CI: 1.88, 8.1.9), had no formal education (RRR = 4.34, 95% CI: 2.19, 8.63), were living in a large family (RRR = 1.82, 95% CI: 1.05, 3.18) and were suffering from non-communicable diseases (RRR = 2.36, 95% CI: 1.32, 4.22) compared to their respective counterparts. The regression analysis also revealed that frailty was significantly higher among participants who were female (RRR = 2.82, 95% CI: 1.34, 5.94), were suffering from non-communicable diseases (RRR = 2.28, 95% CI: 1.27, 4.09), and had feeling of loneliness (RRR = 2.16, 95% CI: 1.11, 4.22).

**Conclusions:**

The findings underscore the need for long-term care and health promotion activities to alleviate the burden of frailty and disability among older adults in humanitarian settings. Efforts should particularly target the most vulnerable groups- older individuals (≥80 years), women, those without formal education, those living in large families, and those with non-communicable diseases.

## Introduction

Ageing populations are steadily growing worldwide, representing a substantial demographic shift. The World Health Organization (WHO) predicts that the world population of older adults aged 60 years and above will increase from 1 billion in 2020 to 2.1 billion by 2050 [1]. Simultaneously, another global demographic shift has been observed through the increased forced migration worldwide [2]. An estimated 123.2 million people were forcibly displaced due to persecution, conflict, violence, and human rights violations [2]. Low- and middle-income countries (LMICs), which already have fragile health and social systems [3] host approximately 73% of the refugees and people in need of protection [4]. Older refugees comprise 4% of the overall refugee population around the globe [5]. Older adults face unique challenges, such as limited access to health care services, reduced social network and quality of support, and limited economic and livelihood opportunities [5,6] in the context of a humanitarian emergency. These challenges are further compounded by age-related physical and physiological phenomena, such as reduced functional ability [7], frailty and disability [8], and cognitive decline [9], which collectively lower the quality of life [10]. Moreover, being overlooked in a humanitarian context, weakened governmental systems, and the absence of safety nets exacerbate the suffering of the older refugees [11]. Bangladesh hosts nearly one million Rohingya refugees from neighboring Myanmar who contribute to the ongoing challenge to the fragile health, economic, social, and environmental system of Bangladesh [12].

Frailty is a multifaceted geriatric syndrome characterised by diminished physiological reserves, decline in cognitive performance and nutritional status, and increased vulnerability to adverse health outcomes [13,14]. Fried and colleagues defined frailty as meeting at least three of the five phenotypic criteria of shrinkage (weight loss), exhaustion, weakness, slow gait speed, and poor physical activity [15]. Disability, on the other hand, refers to difficulties encountered in one or more of the following areas of functioning: impairments (issues with body structure and function), activity limitations (difficulties an individual has performing activities), and participation restrictions (difficulties an individual may encounter when participating in life situations) [16]. Frailty refers to instability and risk of loss of function, while disability indicates loss of function and can often be assessed based on difficulty or dependency in performing activities necessary to live independently [17,18]. Although frailty and disability emerge naturally, their consequences are more severe for older adults in humanitarian contexts due to scarce resources, limited care, and environmental constraints [11].

The Rohingya population, who were forcibly displaced from Myanmar, have experienced state-sponsored persecution, violence, and military attacks in their homeland [19,20]; which has led to waves of mass displacement and forced them to seek refuge in neighboring countries, including Bangladesh [21]. Around one million Rohingyas are currently living in the refugee camp of Cox’s Bazar, Bangladesh, with 52% being children, 44% adults, and 4% older adults [22]. The Rohingya refugee camp is highly populated. As many as 40,000 people live per square kilometer in some areas, making the living standard of the inhabitants one of the worst in the world [23]. The camp’s poor water, sanitation, and hygiene (WASH) condition exacerbate the situation. There is a single communal toilet for every 20 camp residents, often resulting in long waiting times for toilet use [23]. Besides, many toilets are located on higher ground and often lack security lighting, making it difficult for women, children, and older people to access these facilities, especially at night [24]. In line with the ageing population elsewhere, Rohingya older adults experience progressive age-related functional and cognitive decline [7,25]. Rohingya older adults face multifaceted challenges living in Rohingya refugee camp. Evidence indicates that the Rohingya older adults face an array of barriers and challenges in accessing healthcare services [26,27]. Furthermore, the Rohingya older population relies mostly on humanitarian assistance to meet their basic needs, including health and well-being requirements [27,28]. Previous research further provides evidence on the burden of geriatric malnutrition and selected comorbidities among them [29]. The evidence further suggests that displacement-related stressors, such as inadequate access to food, water, shelter, insecure and/or overcrowded living conditions [30], lack of livelihood opportunities [31], and aid dependency [7]` can worsen their quality of life [10] and mental well-being [32]. All these environmental, social, and economic vulnerabilities [33] can exacerbate the onset and consequence of the frailty and disability experience among the Rohingya older adults.

Frailty and disability pose a serious concern among the older population, particularly among migrants and refugees. A study found that immigrants born in low-income countries (LICs) demonstrated a higher frailty index compared to natives and immigrants born in high-income countries (HICs) [34]. Migrants were also found to be frailer than non-migrants in a similar study conducted on 95,635 older adults aged ≥ 50 years living in Europe [35]. The prevalence of frailty among older adults was significantly higher in neighborhoods where a higher proportion of internal migrants reside in Colombia [36]. A three-year follow-up study conducted in 1999 among 534 adult Bosnian refugees originally living in a refugee camp in Croatia revealed that almost half of the older adults had disabilities [37]. Evidence in Africa also suggests that the prevalence of disability is relatively higher among internally and forcibly displaced people [38].

Understanding the unique factors contributing to frailty and disability in this marginalized group is important for developing targeted interventions, improving healthcare provision, and advocating for policy changes that address their specific needs. However, there is a dearth of evidence on frailty and disability among older adults residing in the Rohingya refugee camp in Bangladesh. One study so far found that approximately one in four older adults has limited functional ability [25]. Addressing frailty and disability among the older Rohingya population requires culturally sensitive and context-specific interventions that can account for their unique challenges. Without substantial evidence, designing context-specific interventions targeting this potentially vulnerable Rohingya population would be difficult. The current study, therefore, aimed to examine the prevalence of frailty and disability among the older adults living in Rohingya camp in Bangladesh and to identify factors associated with these conditions.

## Materials and methods

### Study design and participants

This study adopted a cross-sectional design and was conducted between 1 November and 20 December 2021. Data were collected from participants aged ≥60 years who resided in five selected sub-camps in the Rohingya refugee camp in Bangladesh, where a national non-governmental organisation (NGO) operates. The sample size (n = 973) was calculated based on a 50% prevalence (unknown), a margin of error of 5%, a confidence level of 95%, a test power of 80%, and an anticipated 25% non-response rate. Out of the 973 individuals who were initially approached, a total of 864 participants provided their consent to actively participate in this study, yielding a response rate of 88.8%. The NGO had access to a comprehensive registry listing all beneficiaries aged 60 years and above residing within the designated five sub-camps. This exhaustive registry served as the sampling frame for the study. A simple random sampling technique was employed using this sampling frame to select the targeted number of participants. The age of the participants was verified using the SMART cards, which were issued to individuals residing within the camp by the United Nations High Commissioner for Refugees (UNHCR). The inclusion criteria were having aged 60 years and above. Exclusion criteria were suffering from any adverse mental health conditions, i.e., clinically diagnosed schizophrenia, bipolar mood disorder, dementia/cognitive impairment, and inability to communicate.

### Measures

#### Outcome measure.

The primary outcomes of interest were frailty and disability, measured using the ‘Frail Non-Disabled (FiND) questionnaire’ [39]. This questionnaire was previously used among the older adults in Nepal [14], showcasing its relevance and applicability in the South Asian context. However, the FiND questionnaire has not been previously validated in the Rohingya population. For this study, the instrument was translated into the local language used in the camp to ensure comprehension. No additional cultural adaptation or population-specific validation was performed; scoring and classification followed the developers’ algorithm.

The FiND questionnaire comprises five items encompassing a comprehensive evaluation of various facets. The first two questions are on mobility and belong to the disability, and the latter three questions are on weight loss, exhaustion, and physical activity, and belong to the frailty. Each item is coded as “0” for negative responses and “1” for affirmative responses. Participants who respond affirmatively to at least one of the two disability items are classified as “disabled”, respond negatively to both disability questions but affirmatively to at least one frailty question are classified as “frail”, and respond negatively to each of the five items are classified as “robust”, indicating the absence of both frailty and disability. Cronbach’s α was calculated to determine the internal consistency and reliability of the FiND questionnaire within the targeted study population. The calculation produced a Cronbach’s α value of 0.75, indicating an acceptable level of reliability [40].

#### Explanatory variables.

The explanatory variables were selected through an extensive review of pertinent literature [14,35,41–43]. Age in years was stratified into three distinct categories: 60–69, 70–79, and ≥80, allowing for a comprehensive examination of age-related nuances. Sex was dichotomously classified as male or female, enabling an exploration of potential gender disparities. Marital status was categorised into two groups: currently married or without a partner, providing insights into the influence of intimate relationships on the outcomes. The variable of formal education was dichotomized as no or yes, providing an understanding of the potential impact of educational attainment. Family size was also dichotomised as ≤4 or >4, allowing for an exploration of the influence of household composition on the outcomes. Monthly family income was categorized as living with aid only, < 5000 BDT, and ≥5000 BDT, while occupation was classified as eployed or unemployed/retired. Participants were also categorized as either having a self-repoted memory or concentration problems or not. Living arrangements were categorised as either living alone or with family, shedding light on the potential consequences of available familial support. Level of physical activity was categorized as either ‘having regular physical activity’ or ‘none or mainly sedentary’. The information on the presence of non-communicable diseases was collected through asking the participants if they have ever been diagnosed with arthritis, hypertension, heart disease, stroke, hypercholesterolemia, diabetes, chronic respiratory diseases, chronic kidney disease, or cancer by a heathcare provider. Participants were then categorized as either suffering from any non-communiable diseases or not.

The assessment of loneliness was undertaken utilizing the 3-item UCLA Loneliness Scale [44], a well-established tool previously employed among the Bangladeshi population [45]. This scale comprises three distinct items, each evaluated through a yes/no format. The three items encompass the following aspects: (i) the frequency of experiencing a lack of companionship, (ii) the frequency of feeling left out, and (iii) the frequency of feeling isolated from others over the preceding two-week period. Each item within the scale was measured using a 3-point Likert response scale: hardly ever (1 point), some of the time (2 points), and often (3 points). Participants were subsequently classified as experiencing loneliness if they responded with “some of the time” or “often” to any of the items [46].

### Data collection tools and techniques

A semi-structured questionnaire was employed to collect pertinent information. Data were collected using the SurveyCTO mobile application (https://www.surveycto.com/) and was executed by twenty-two local enumerators. These individuals, hailing from the local community, displayed profound fluency in Rakhine dialects and had prior experience in proficiently executing health-related surveys using electronic platforms. Before starting data collection, the enumerators were trained thoroughly by the research team to acquaint them with data collection tools and techniques.

The English version of the questionnaire was translated into the Rakhine language, and then back-translated into English to check if the intricacies of the source items were properly preserved. Two local NGO personnel proficient in English and the Rakhine languages assisted in this process. After developing the Rakhine version through cross-checking and resolving minor disagreements about the translation, the draft Rakhine instrument was piloted with a small sample (n = 10) of Rohingya older adults from the camp. The pilot administration of the instrument helped further refine the language in the final version of the questionnaire. The data was collected using this final tool through face-to-face interviews of the participants. Each interview took approximately half an hour to complete. The English version of the final questionnaire is provided as S1 File.

### Statistical analyses

Descriptive analyses (i.e., frequency and percentage) were performed to explore the distribution of the participants in terms of sociodemographic variables. Considering the fact that outcome variable is categorical with more than two unordered responses, a multinomial logistic regression model was performed [47] to identify the factors independently associated with frailty and disability while controlling for important confounders. The covariates to be included in the final regression model were determined using a backward elimination approach guided by the Akaike information criterion (AIC) [48]. The initial model included all potential covariates outlined in Table 1. In the next step, variables were sequentially removed based on their AIC value and concomitant statistical significance to achieve the final sets of variables for a parsimonious model. The final regression model was performed, including these variables. The Relative Risk Ratio (RRR) and its corresponding 95% confidence interval (95% CI) for the “disabled” and “frail” categories are provided with reference to the “robust” category in Table 2. As the multinomial regression model estimates probabilities rather than odds, and that odds ratio could overestimate the associations, as the outcomes were common, we used RRR instead of odds ratio (OR) to measure association [47]. All statistical analyses were conducted using the Stata software package (Version 14.0).

**Table 1:** Characteristics of the participants (N = 864).

Characteristics	N	%
Age (year)		
60–69	625	72.34
70-79	190	21.99
≥ 80	49	5.67
Sex		
Male	486	56.25
Female	378	43.75
Marital status		
Married	683	79.05
Without partner	181	20.95
Formal schooling		
No formal schooling	769	89.00
Having formal schooling	95	11.00
Family size		
≤ 4	372	43.06
> 4	492	56.94
Family monthly income (BDT)		
Living on aid only	580	67.13
< 5000	220	25.46
≥ 5000	64	7.41
Current occupation		
Employed	94	10.89
Unemployed/retired	770	89.12
Living arrangement		
Living with family	782	90.51
Living alone	82	9.49
Problem with memory or concentration		
No problem	363	42.01
Memory or concentration problems	501	57.99
Suffering from non-communicable diseases		
No	431	49.88
Yes	433	50.12
Level of physical activity		
Regular physical activity	295	34.14
None or mainly sedentary	569	65.86
Feeling of loneliness		
No	163	18.87
Yes	701	81.13

**Table 2:** Bivariate analysis for frailty and disability (N = 864).

Characteristics	Robust	Disabled	Frail
Overall	%	%	%
	7.87	55.21	36.92
Age (year)			
60–69	9.28	50.40	40.32
70-79	4.74	63.68	31.58
≥ 80	2.04	83.67	14.29
Sex			
Male	11.52	49.79	38.68
Female	3.17	62.17	34.66
Marital status			
Married	8.64	52.56	38.80
Without partner	4.97	65.19	29.83
Formal schooling			
No formal schooling	6.37	58.00	35.63
Having formal schooling	20.00	32.63	47.37
Family size			
≤ 4	8.06	46.51	45.43
> 4	7.72	61.79	30.49
Family monthly income (BDT)			
Living on aid only			
< 5000	5.34	61.72	32.93
≥ 5000	9.38	53.12	37.50
Current occupation			
Employed	23.40	29.79	46.81
Unemployed/retired	5.79	58.31	35.71
Living arrangement			
Living with family	8.18	54.73	37.08
Living alone	4.88	59.76	35.37
Problem with memory or concentration			
No problem	12.40	35.26	52.34
Memory or concentration problems	4.59	69.66	25.75
Suffering from non-communicable diseases			
No	10.44	52.20	37.35
Yes	5.31	58.20	36.49
Level of physical activity			
Regular physical activity	23.05	28.47	48.47
None or mainly sedentary	0.00	69.07	30.93
Feeling of loneliness			
No	11.04	60.12	28.83
Yes	7.13	54.07	38.80

### Ethics approval and consent to participate

The study was approved by Jashore University of Science and Technology’s institutional review committee (Ref: ERC/FBST/JUST/2020–61). As participants were recruited from a forcibly displaced, vulnerable population, additional considerations were taken to ensure the participation of the older adults. Participants were thoroughly apprised of the study’s objectives in their preferred languages before seeking their informed consent. It was also ensured that their consent and participation were not influenced by the aid providers. Participation was voluntary, and participants did not receive any compensation for their time.

### Patient and public involvement

Patients and/or public were not involved in the development of the research question, study design, conducting study and result dissemination.

## Results

### Characteristics of the participants

The sociodemographic profiles of the study participants are outlined in Table 1. A substantial proportion of the respondents were within the age range of 60–69 years, accounting for 72.34% of the sample, 21.99% were between 70–79 years old, and 5.67% were aged 80 years or above. Male respondents were a notable majority, representing 56.25% of the participants. Most participants (79.05%) were married and 56.94% resided in households comprising four or more individuals. A large portion of the participants (67.13%) relied solely on external assistance for sustenance. Approximately 89.00% of the participants were unemployed or retired, demonstrating the prevalence of reliance on aid alone and the lack of employment opportunities. Most individuals (90.51%) in the sample indicated that they lived with family, and most (89.00%) had no formal education.

More than half of the participants exhibited diminished memory or concentration, amounting to around 58.00% of the sample, while half (50.12%) suffered from non-communicable diseases, signifying the prevalence of such health conditions within the population under investigation. Additionally, a majority of the sample, encompassing 65.86% of the participants, reported a lack of regular physical activity. Remarkably, a staggering 81.13% of the participants reported experiencing feelings of loneliness, emphasizing the prevailing sense of isolation among the sampled individuals.

### Prevalence of frailty and disability

Table 2 outlines the prevalence of non-disabled frailty and disability across different characteristics of the participants to provide descriptive context and identify crude patterns that inform the specification of our multivariable models. Data revealed that a substantial portion (36.92%) of the entire cohort (n = 864) exhibited frailty, while an even higher portion (55.21%) of them grappled with a disability, leaving a mere 7.87% classified as robust. Notably, the prevalence of frailty was relatively higher among individuals aged 60–69 years compared to those aged 80 years and above (40.32% versus 14.29%). Conversely, the prevalence of disability significantly affected the latter group, plaguing 83.67% of those aged 80 years and above compared to 50.40% among their counterparts aged 60–69 years. Furthermore, bivariate analysis revealed that males demonstrated a higher incidence of frailty (38.68%) compared to females (34.67%), whereas females showed a higher rate of disability (62.17%) compared to males (49.79%).

The bivariate analysis also suggested that respondents who did not have any formal education had a higher prevalence of disability (58.00%) than frailty (35.63%) and participants with some formal schooling demonstrated a higher prevalence of frailty (47.37%) compared to disability (32.63%). In addition, participants who had four or fewer family members had a slightly higher prevalence of disability (46.51%) compared to frailty (45.43%), while participants with more than four family members had relatively higher prevalence of disability (61.79%) than frailty (30.49%).

### Factors associated with frailty and disability

Table 3 illustrates the independent factors associated with frailty and disability in the multinomial logistic regression model. The analysis revealed compelling evidence that the likelihood of experiencing disability was markedly higher among participants in the age bracket of 70–79 years (RRR = 2.65; 95% CI: 1.25, 5.66) and those aged 80 years or older (RRR = 8.06; 95% CI: 1.05, 61.80), as well as among females (RRR = 3.93; 95% CI: 1.88, 8.19), those deprived of formal education (RRR = 4.34; 95% CI: 2.19, 8.63), those from families consisting of four or more members (RRR = 1.82; 95% CI: 1.05, 3.18), and those suffering from non-communicable diseases (RRR = 2.36; 95% CI: 1.32, 4.22), as compared to their respective counterparts.

**Table 3:** Factors associated with disability and frailty in comparison to robustness (N = 864).

Characteristics	Disabled			Frail		
	RRR^1^	95% CI	*P*	RRR	95% CI	*P*
Age (year)						
60–69	*Ref*					
70-79	2.65	1.25, 5.66	**0.011**	1.61	0.74, 3.47	0.229
≥ 80	8.06	1.05, 61.80	**0.045**	1.67	0.19, 14.26	0.635
Sex						
Male	*Ref*					
Female	3.93	1.88, 8.19	**<0.001**	2.82	1.34, 5.94	**0.006**
Marital status						
Married	*Ref*					
Without partner	0.87	0.38, 1.99	0.748	0.68	0.29, 1.58	0.366
Formal schooling						
Having formal schooling	*Ref*					
No formal schooling	4.34	2.19, 8.63	**<0.001**	1.87	0.98, 3.57	0.058
Family size						
≤ 4	*Ref*					
> 4	1.82	1.05, 3.18	**0.034**	0.84	0.78, 1.47	0.564
Living arrangement						
Living with family	*Ref*					
Living alone	1.47	0.51, 4.57	0.507	1.07	0.34, 3.36	0.911
Suffering from non-communicable diseases						
No	*Ref*					
Yes	2.36	1.32, 4.22	**0.004**	2.28	1.27, 4.09	**0.006**
Feeling of loneliness						
No	*Ref*					
Yes	1.34	0.70, 2.57	0.377	2.16	1.11, 4.22	**0.024**

^1^RRR = Relative Risk Ratio; Note: All variables were considered together in this adjusted model

Conversely, the odds of frailty were notably higher among females (RRR = 2.82, 95% CI: 1.34–5.94), individuals grappling with non-communicable diseases (RRR = 2.28, 95% CI: 1.27–4.09), and those who experienced a sense of loneliness (RRR = 2.16, 95% CI: 1.11–4.22).

## Discussion

This study examined frailty and disability among the older population residing in the Rohingya refugee camp in Bangladesh. The findings revealed that the prevalence of frailty and disability among older adults was 36.9% and 55.5%, respectively. Notably, no previous research reported the frailty and disability among older adults in Rohingya camp in Bangladesh. However, the prevalence of frailty among Rohingya older adults surpasses that of older adults residing in rural Tanzania, as demonstrated by a previous study [49]. It is also higher than another study conducted in the United States, where the prevalence of frailty was 15% among older refugees [50]. A study conducted on forced and internally displaced older adult migrants in Colombia unveiled an alarming prevalence of pre-frailty/frailty, estimated at 80.63% [36]. Another study conducted on Bosnian older refugees reported a disability prevalence of 46% [37] which is close to the estimates found in the present study. The Global AGEing and Adult Health (SAGE) Wave 1 study indicated that both frailty and disability prevalence were highest in India at 55.5% and 93.3% respectively among older adults [41]. A study conducted in Saudi Arabia also unveiled that frailty and pre-frailty affected 21.4% and 47.3% of the population, respectively [51].

The higher prevalence of frailty and disability among Rohingya older adults can plausibly be ascribed to their limited access to fundamental healthcare services, inadequate nutritional support and outcomes, substandard water, sanitation, and hygiene (WASH) provisions, overcrowded living conditions, limited or absent social protection, insufficient care, and meager or absent financial assistance [23,24,52,53]. Moreover, older adults often face exclusion from different services and a dearth of programs specifically tailored to their needs, offered by humanitarian organizations [26,54], which can contribute to this high prevalence. The heightened prevalence of frailty and disability underscores the increased vulnerability of this population to negative health outcomes [55], diminished quality of life [56], and feelings of isolation [57].

The current study highlighted that the prevalence of frailty was higher among individuals aged 60–69, while the occurrence of disability was notably more pronounced in the ≥ 80 age group compared to those in the 60–69 age range. The aging process entails a multitude of physical, psychological, social, and environmental modifications that contribute to the emergence of frailty among older individuals [58]. Consequently, disability, serving as a deleterious consequence resulting from frailty [59], intensifies with advancing age [60,61].

The findings of our research suggest a propensity for heightened levels of frailty and disability in female participants compared to their male counterparts. This finding aligns with comparable research conducted across diverse settings [62–64]. Factors such as longevity, a higher incidence of chronic diseases, and a confluence of social, economic, and health disparities may render females more susceptible to frailty and disability than males [62,64]. The vulnerable position of females within the Rohingya community is accentuated by limited access to crucial health and ancillary services, inadequate provisions for clean water and sanitation, and socio-cultural constraints that curtail women’s ability to access life-saving resources and practices [24,65]. These factors could plausibly account for the increased prevalence of frailty and disability observed among older women within the Rohingya refugee camp.

Our study also found a negative correlation between formal educational attainment and the prevalence of frailty among older adults. This finding mirrors analogous observations made across divergent settings [42,43,66,67]. Formal education, alongside other socioeconomic, cultural, and demographic factors, profoundly influences disease determinants, thereby influencing the onset of maladies throughout one’s lifespan. Education further engenders health-seeking behaviors, care practices, utilization of medical interventions, and the adoption of assistive devices in the face of frailty and disability [68]. Similar to the findings of this study, previous studies also showed that the level of formal schooling is low among older adults [64,69]. In populations with high illiteracy, particularly in refugee camps, formal education may function more as an indicator of early-life socioeconomic advantage than as a direct influence on health-related decision-making [70]. Older adults with some educational attainment may have benefited from better living conditions, nutrition, and healthcare access throughout the life course, factors known to delay vulnerability and frailty [71]. Consequently, the observed protective effect of education may reflect cumulative socioeconomic advantage rather than literacy-related behavioural differences [72]. Moreover, in resource-limited and structurally constrained environments, the capacity to translate education into informed health choices is often restricted by limited service availability and systemic barriers [73,74]. In such settings, material and social resources may play a more decisive role in shaping late-life health outcomes, consistent with life-course frameworks emphasizing the long-term impact of social conditions on ageing and frailty [75,76].

The present study also found that older adults living in a large family had higher levels of disability than those living in a small family. This is probably because large families usually have a higher poverty level [77], and older family members receive less care from family members who are more concerned about their livelihoods [78]. However, while statistically insignificant, prevalence of frailty was lower among older adults from large families, which need to be interpreted cautiously. Conceptually, disability and frailty capture related but distinct constructs, task-specific functional limitation versus physiological reserve. In our setting, larger multigenerational households often co-occur with lower socioeconomic resources and greater caregiving demands. These factors can increase the observed prevalence of disability (e.g., limitations in ADLs/IADLs) through crowding, injury risk, infection exposure, or earlier recognition/reporting of functional limitations by co-residents. At the same time, frailty may be buffered by the greater instrumental and emotional support available in larger families (assistance with meals, mobility, medication management, and social engagement), which can mitigate weight loss, inactivity, and exhaustion—core frailty components—even if some functional disabilities are present. This pattern is consistent with the notion that multigenerational support may ameliorate frailty features even where functional limitations persist or are more readily recognized.

This study has also demonstrated the correlative relationship between non-communicable diseases and an escalated state of frailty and disability. This finding is further supported by the aggregated empirical data originating from diverse geographical contexts across the globe [42,69]. Researchers have recurrently highlighted an elevated occurrence of non-communicable diseases among refugees and asylum seekers, attributable to transformative modifications in their manner of living, such as their dietary inclinations and physical exertion [79–81]. Rohingya older adults, with their inherent vulnerabilities and sociopolitical disadvantages, are predisposed to the manifestation of non-communicable diseases [82]. Prior inquiries into older Rohingya cohorts have corroborated the augmented prevalence of enduring non-communicable diseases in this demographic [25,83], thereby elucidating the amplification of frailty and disability observed amongst older adults. We also found that those who felt lonely had higher frailty. Loneliness imparts negative emotions and distress, interfering with the performance of daily activities [83].

### Implications for policy and practice

The findings of this study underscore the imperative for a cohesive and collaborative approach among stakeholders, authorities, and partners in order to safeguard the health and well-being of older individuals dwelling in refugee settings. At every juncture of program design and execution, stakeholders and authorities must duly consider the distinct needs of older adults. While multiple agencies are engaged in the Rohingya refugee camp, only a few organizations such as Young Power in Social Action (YPSA), Help Age International-Bangladesh, Center for Disability in Development (CDD), Handicap International, the International Organization for Migration (IOM), direct their efforts toward the welfare of the older adults [84,85]. Organizations dedicated to the welfare of the older population should unite and pool their resources with a steadfast focus on catering to the specific needs of this demographic. While numerous programs operate on a large scale, encompassing all sub-camps in the Rohingya response in Bangladesh, programs designed specifically for older adults remain limited in scope. Thus, it is incumbent upon donor agencies, implementing organizations, and other pertinent stakeholders to proactively address this high burden of frailty and disability among this vulnerable population.

### Strengths and limitations of the study

The current study has several strengths and limitations. No previous study has examined frailty and disability, and the associated factors affecting Rohingya older adults. Thus, our study will contribute to the existing body of literature concerning frailty and disability within the displaced, migrant, and refugee populations. However, this study is not without its limitations. Firstly, implementing a cross-sectional design imparts a momentary glimpse into the subject matter, precluding the establishment of causal relationships. Second, the study was confined to a selected number of sites due to constraints imposed upon the capacity for data collection, which poses the question of its generalization for the entire Rohingya camp. Third, a key limitation is the absence of a formal cultural adaptation/validation of FiND for the Rohingya context beyond language translation. While FiND is designed for brief community screening and has been used in regional settings, population-specific validation (e.g., cognitive interviews, test–retest reliability, and construct validity against clinical assessments) is warranted and planned in future work. Fourth, use of self-reported health-related data can further lead to underreporting or misclassification of the non-communicable diseases, particularly by participants with low educational level, limited access to health care services, and limited health literacy. Some chronic disease symptoms can be misclassified, unrecognized, or under-reported. This can impact the estimation of the true burden of non-communicable diseases. Self-reporting can also induce social desirability bias and recall bias, which are important limitations to consider. Finally, frailty comparisons may have been underpowered after stratification; future work should evaluate these relationships longitudinally and with population-specific validation.

## Conclusion

The current study revealed a high prevalence of both frailty and disability among older adults in the Rohingya refugee camp in Bangladesh. Our study findings highlight the vulnerability of older adults to frailty and disability and indicate related vulnerability factors, including higher age, female sex, not having formal education, living in large families, suffering from non-communicable diseases, and loneliness. The implications of this study emphasize the need for collaborative efforts among stakeholders to deliver long-term care and health promotion initiatives to reduce the higher level of frailty and disability among the older population. In accordance with the findings of the research, it is also recommended that these interventions be particularly focused on the most vulnerable segments of the population mentioned above.

## Supporting information

S1 File English version of the questionnaire for data collection. (DOCX)

S2 File Dataset of the study. (XLS)
