# Supplementary material for: Frailty and disability among older adults residing in Rohingya refugee camp in Bangladesh
Source: PLoS One. 2026 Jan 23;21(1):e0341499. doi: 10.1371/journal.pone.0341499 (PMC12829860; doi:10.1371/journal.pone.0341499)
Supplement: S1 File — (DOCX) [file pone.0341499.s001.docx]

**Frailty and disability among older adults in Rohingya refugee camp**

**Questionnaire**

| Household ID: \|__\|__\|__\|__\| | Date: |
| --- | --- |
| Respondent’s name: | Father’s name: |
| Village/Camp: | Union: |
| Upazila: | Mobile: |

# Respondent’s socio-demographic characteristics

| **Sl No** | **Questions** | **Answer** | **Code** | **Skip to** |
| --- | --- | --- | --- | --- |
|  | How old are you now? | (Years) |  |  |
|  | Sex of respondent: | Male  Female |  |  |
|  | What is your current marital status? | Married  Widow/Widower  Divorced  Separated  Unmarried |  |  |
|  | Did you receive any formal schooling? | Yes  No | 1  2 |  |
|  | Family size |  |  |  |
|  | What is the current monthly income of your household? | BDT |  |  |
|  | What is your current main occupation? | Housewife  Household based work (weaving, handicraft)  Maid  Skilled labor (carpenter, goldsmith etc.)  Day laborer  Driver (truck, bus)  Rickshaw/van puller etc.  Garments worker  Business (Big)  Service  Small business (milkman, fruit, vegetable seller etc.)  Unemployed/ Retired  Agriculture  Disable  Others, specify:  Don’t know | 1  2  3  4  5  6  7  8  9  10  11  12  13  14  -96  -99 |  |
|  | Living arrangement | Alone  With family members/others | 1  2 |  |
|  | Having problems with memory or concentration during last month preceding the survey? | Low memory or concentration  No problem | 1  2 |  |

# Morbidity

|  |  | Are you currently suffering from any chronic disease?  [ENUMERATOR: Ask for each of the disease separately]  Code:   1. Yes 2. No 3. Don’t know | 2. Are you taking medication for this?  Code:   1. Yes 2. No 3. Don’t know |
| --- | --- | --- | --- |
|  | Arthritis |  |  |
|  | Hypertension |  |  |
|  | Heart disease |  |  |
|  | Stroke |  |  |
|  | Raised total cholesterol |  |  |
|  | Diabetes |  |  |
|  | Chronic lung disease |  |  |
|  | Chronic kidney disease |  |  |
|  | Cancer |  |  |

# Frail Non-Disabled (FiND) Questions

|  | Have you any difficulties at walking 400 meters? | 0. No or some difficulties  1. A lot of difficulties or unable |
| --- | --- | --- |
|  | Have you any difficulties at climbing up a flight of stairs? | 0. No or some difficulties  1. A lot of difficulties or unable |
|  | During the last year, have you involuntarily lost more than 4.5 kg? | 0. No  1. Yes |
|  | How often in the last week did you feel that everything you did was an effort or that you could not get going? | 0. Rarely or sometimes (2  times or less/week) 1. Often or almost always (3 or  more times per week) |
|  | Which is your level of physical activity? | 0. Regular physical activity (at  least 2-4 hours per week)  1. None or mainly sedentary |

**Thank you!**
